# Innovative oleogels: Developing sustainable bioactive delivery systems for healthier foods production

**DOI:** 10.1016/j.fochx.2025.102768

**Published:** 2025-07-09

**Authors:** Taha Mehany, Oscar Zannou, Kouame F. Oussou, Ifagbémi B. Chabi, Reza Tahergorabi

**Affiliations:** a Food Technology Department, Arid Lands Cultivation Research Institute, City of Scientific Research and Technological Applications (SRTA-City), 21934 New Borg El Arab, Alexandria, Egypt; b Department of Food Technology, Vocational School of Technical Sciences at Mersin Tarsus Organized Industrial Zone, Tarsus University, 33100 Mersin, Turkey; c Laboratory of Human Nutrition and Valorization of Food Bio-Ingredients, Faculty of Agricultural Sciences, University of Abomey-Calavi, Cotonou 01 BP 526, Benin; d Department of Food Engineering, Faculty of Agriculture, Çukurova University, 01330 Adana, Turkey; e Food and Nutritional Sciences Program, North Carolina A&T State University, Greensboro, NC 27411, USA

**Keywords:** Oleogel, Encapsulation, Oleogelation, Oleogel drug delivery, Omega fatty acids, Bioavailability, Bioaccessibility, Nutritional delivery, Proteins, Fat replacers

## Abstract

Oleogels have emerged as an excellent medium for delivering bioactive compounds. This study highlights and discusses recent advances in developing, producing, characterizing, and applying oleogels for delivering functional and bioactive compounds in food products. The review revealed that oleogels present a promising opportunity to create healthier food products by enhancing their functional and nutritional profiles. Oleogels have become one of the most effective encapsulation and delivery systems for various bioactive molecules, including polyphenols, omega fatty acids, vitamins, essential amino acids, natural essential oils, and proteins. Oleogels showed an efficiency in protecting bioactive compounds from degradation within the digestive tract. They have successfully reformulated foods, such as bakery and meat products, with acceptable or even superior techno-functional and physicochemical properties. However, significant challenges remain in improving the processing, formulation, and oleogelation techniques. Further research is strongly recommended to address the reviewed limitations and expand the industrial applications.

## Highlights

• Oleogels offer a healthier alternative to traditional solid fats in food formulations.
• They can replace milk fat in dairy products and fats in meat-based products.
• Oleogels are promising for microencapsulation and delivery of lipid-soluble bioactive compounds.
• They protect sensitive bioactives during digestion, improving stability and bioavailability.
• Challenges such as scalability and consumer acceptance require further research.

## Graphical abstract

**Figure:**
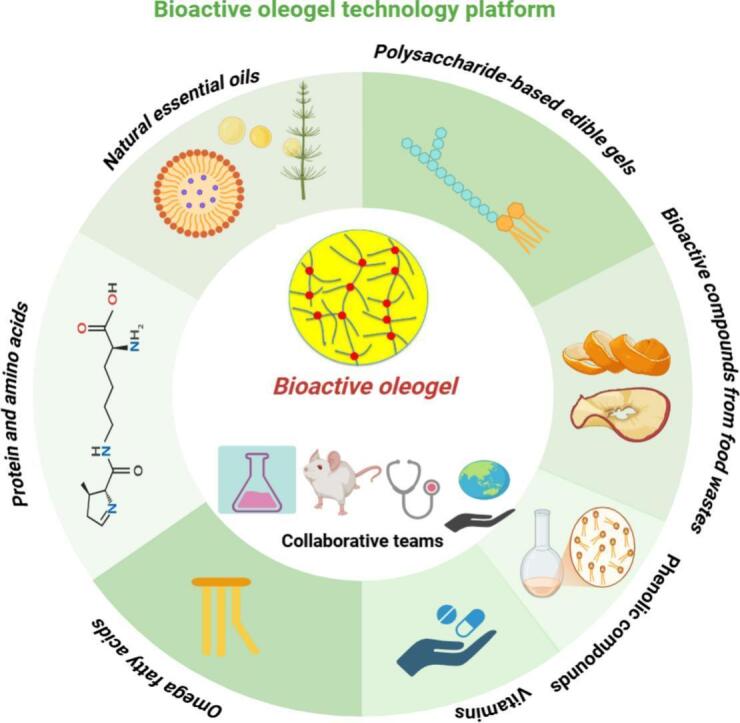

## 1 Introduction

The physicochemical properties of solid fats make them widely applicable in the food, cosmetic, and pharmaceutical industries. In the food sector, solid fats, known for their high content of saturated fatty acids (SFAs), impart desirable organoleptic properties to food products, particularly in terms of texture, flavor, spreadability, binding, mouthfeel, and functionality (Barroso et al., 2022; Manzoor et al., 2022; Pușcaș et al., 2020). However, excessive consumption of trans and SFAs is strongly linked to cardiovascular diseases and other metabolic disorders, including obesity and type 2 diabetes (Issara et al., 2020; Martins et al., 2018; Oteng & Kersten, 2020). Many researchers emphasize that the health risks associated with high intake of trans and SFAs can only be mitigated by reducing their presence in food products and diets (Ahmed et al., 2018; Oteng & Kersten, 2020). Consequently, reducing or replacing trans and SFAs in the diet has become a significant challenge for both industrial and scientific communities. To address this, various strategies have been explored to replace trans and saturated fats with healthier bio-based ingredients, such as carbohydrate fatty acid polyesters, gums, polydextrose, starch, cellulose, whey, lactoglobulins, egg white, soy, and others (Pușcaș et al., 2020). However, a common shortcoming of these alternatives is their inability to replicate the sensory properties of traditional solid fats, which often fails to meet consumer expectations and leads to dissatisfaction with the final product (Issara et al., 2020; Pușcaș et al., 2020).

Furthermore, the interesterification technique, considered a promising approach for replacing trans fatty acids, requires extensive chemical treatments to achieve the desired physicochemical characteristics (Barroso et al., 2022). Additionally, concerns about its potential adverse health effects have limited its widespread adoption (Bihola et al., 2025; Dhiman et al., 2024).

Recently, emulsion gel systems have garnered significant attention as a promising alternative for solid fat replacement in the food industry, aiming to mitigate the adverse effects of solid fats on human health. An emulsion gel, also known as an emulgel or gelled emulsion, is a complex soft material composed of both gels and emulsion droplets (Abdullah et al., 2022; Lin et al., 2020; Shakeel et al., 2019). These systems are particularly valuable due to their ability to mimic the physicochemical properties of conventional solid fats while allowing the incorporation of healthier lipids such as unsaturated fatty acids and functional bioactive compounds (Dickinson, 2013; Lu et al., 2019).

Recent studies have explored a wide range of biopolymers and structuring agents—such as proteins (e.g., soy protein isolate, whey protein), polysaccharides (e.g., pectin, alginate, carrageenan), and their combinations—to formulate stable and functional emulsion gels (Fontes-Candia et al., 2023; Lin et al., 2021). Emulsion gels have shown great potential in applications such as meat analogues, bakery products, and dairy foods, where they contribute to desired textural and sensory attributes while improving the lipid profile of the final products (Kothuri et al., 2024; Xu et al., 2024).

Moreover, emulsion gels can be tailored to deliver lipophilic nutrients and bioactive compounds, enhancing their stability and bioavailability, thus contributing to the development of functional foods (Li et al., 2023). Their dual structure allows for better control over digestion and lipid release, which is particularly beneficial in the design of satiety-inducing or low-fat food products (Li et al., 2022).

By definition, an oleogel consists of at least two components:

(i) the gelator (or gelling agent), which exhibits reduced mobility, and

(ii) the solvent, which behaves like a liquid. The chemical nature of the solvent (polar or non-polar) determines two main categories of emulsion gels: hydrogels (for aqueous phases) and organogels (for organic phases or non-polar solvents). When the organic phase is an edible oil, the organogel is referred to as an oleogel. In food and pharmaceutical applications, edible oils such as canola oil, olive oil, and sunflower oil are commonly used as solvents for oleogel preparation (Issara et al., 2020; Shakeel et al., 2019).

Furthermore, a gel composed of two different gels is called a bigel. Essentially, a bigel is a semi-solid biphasic system formed from two distinct gels. In this context, a bigel combines gels with different polarities, typically a hydrogel and an oleogel. The oleogel may serve as the dispersed phase while the hydrogel forms the continuous phase, or vice versa (Shakeel et al., 2019; Xie, Hu, et al., 2023). The resulting bigel integrates the key properties of hydrogels and oleogels, making it a versatile system for delivering both lipophilic and hydrophilic agents. A variety of gels have been developed, including hydrogels (Light & Karboune, 2022), organogels (Li & Liu, 2023; Manzoor et al., 2022), aerogels (Dhua et al., 2022), cryogels, xerogels, and bigels (Pascuta et al., 2022; Xie, Hu, et al., 2023), each with specific techniques and unique features. Among these, organogels, particularly edible oleogels, stand out as the most popular choice for replacing trans and saturated fatty acids, offering a wide range of applications in the food industry (Martins et al., 2018). The growing interest in oleogel development within the food sector can be attributed to its: (i) biocompatible nature as a solid fat replacement, (ii) versatile techno-functional properties, and (iii) health and wellness-promoting capabilities (Barroso et al., 2022; Martins et al., 2018). Oleogel is an effective bio-ingredient for mimicking the physical and sensory properties of solid fats. It enhances the stability, bioavailability, and shelf life of food products (Dhiman et al., 2024; Bihola et al., 2025). Notably, Issara and colleagues demonstrated that oleogels composed of beeswax (gelator) and canola oil exhibited significant anti-adipogenesis activity and promote angiogenesis in obese rats (Issara et al., 2020). In addition, the oleogel containing bioactive compounds showed strong cytotoxic effects against colorectal Caco-2 cancerous cells (Akl et al., 2025).

**Fig. 1:**
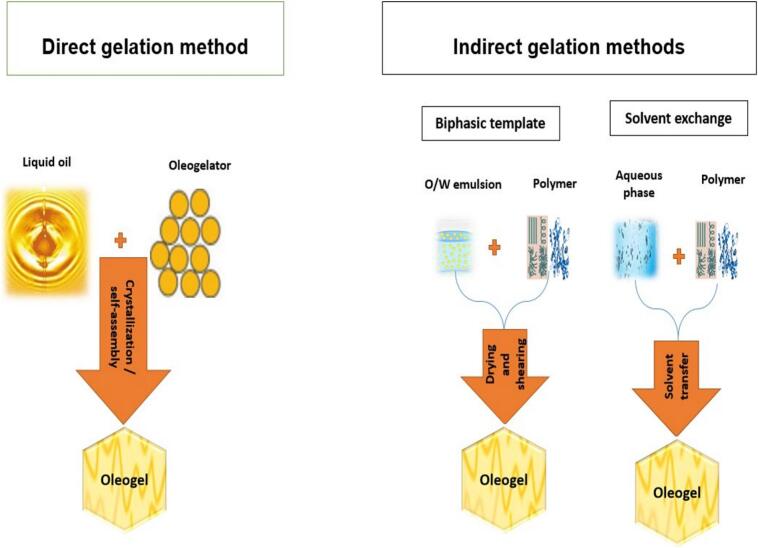
Illustration of approaches for oleogels formation.

Oleogels can be prepared using two main approaches (1) Indirect techniques, such as the emulsification process and solvent exchange (Barroso et al., 2022; Manzoor, Masoodi, Naqash, & Rashid, 2022), (2) Direct techniques, which involve directly mixing the oleogelator with oil at a temperature above the gelator's melting point, followed by cooling (Barroso et al., 2022; Zhao et al., 2022). Fig. 1. illustrates of oleogels formation approaches. Additionally, it is possible to formulate oleogels with tailored properties depending on the specific physical characteristics required and the type of food application (Martins et al., 2018; Zhang et al., 2024). Beyond food applications, oleogels have also proven to be excellent vehicles for delivering bioactive compounds. This study highlights and discusses the most recent advances in the development, production, characterization, and application of oleogels for delivering functional and bioactive compounds in food systems.

## 2 Oleogels opportunities to formulate healthier foods

Oleogels present a promising opportunity for producing healthier fried products by enhancing the nutritional composition of the end products (Table 1). These innovative structured oil systems combine liquid oil with structuring agents, resulting in semi-solid or gel-like textures. Incorporating oleogels into food products changes their physical attributes, as the interaction between oleogels, processing conditions, and the external environment can synergistically influence their functionality and the overall quality of food products. Precisely engineered oleogels with enhanced physicochemical properties hold significant potential for formulating foods with textural characteristics comparable to those made with traditional solid fats (Martins et al., 2020). This innovation reduces oil absorption during frying, thereby improving the nutritional profiles of the final products. This progressive approach aligns with the growing demand for healthier food options, particularly in the realm of fried and baked goods. By leveraging oleogels, food manufacturers and scientists can reduce oil content, enhance nutritional value, and create fried products that appeal to health-conscious consumers. This novel strategy highlights the potential of oleogels to revolutionize healthier fried foods, enabling consumers to enjoy the crispy, flavorful experience they love while benefiting from improved nutritional quality.

**Table 1:** Overview of oleogels used in the food industry: a brief description, structuring strategies, and key findings.

**Food product**	**Analyzedoleogels**	**Structuringstrategy**	**The main findings**	**Reference**
Pastry	Sunflower oil was gelled using a structured protein (1–5 wt%) network combined with low concentrations of candelilla wax.	An emulsion-templated method, followed by storage and a drying process.	Microscopy and rheological analyses revealed that the addition of wax significantly enhanced shear recovery and oil binding capacity, without notably affecting gel strength. Practical application in shortcrust pastry doughs showed that these hybrid oleogels structured dough better than wax-based oleogels alone, although not as effectively as traditional vegetable oil-based margarine. Combining oleogelation techniques can synergistically maximize functional benefits and minimize drawbacks, enhancing the feasibility of oleogels as fat replacers in food applications.	(Tavernier et al., 2018)
	An emulsion consisting of oil (47 % w/w) in water (51.5 % w/w), stabilized with hydroxypropyl methylcellulose (1.5 % w/w).	Emulsion-templated method followed by evaporation of the aqueous phase at 60 °C.	Replacing shortening with oleogel resulted in croissants with reduced saturated fat content, decreased bite firmness, and a texture profile comparable to that of croissants made with commercial shortening.	(Espert et al., 2023)
	Sunflower oil was gelled using sodium stearoyl lactylate at concentrations of 7 %, 9 %, 11 %, and 13 % (w/w).	Direct dispersion, followed by cooling at 20 °C for 24 h.	Sodium stearoyl lactylate (SSL) effectively structured sunflower oil into oleogels, with higher concentrations (7–13 % w/w) leading to denser crystal networks. These denser structures enhanced solid fat content, oil retention, and firmness by mimicking the functionality of triacylglycerol crystalline networks. The improved mechanical strength was linked to SSL crystal interactions, including van der Waals forces and electrostatic repulsion, as confirmed by polarizing light microscopy (PLM), X-ray diffraction (XRD), rheology, differential scanning calorimetry (DSC), and Fourier transform infrared spectroscopy (FTIR).	(Meng et al., 2019)
	Ethyl cellulose and adipic acid were used to gel soybean oil at different concentrations (0–6 wt%).	Direct dispersion, followed by cooling to ambient temperature.	- Adipic acid improved the thermal behavior and crystallinity of ethyl cellulose-based oleogels. - The 2 % ethyl cellulose/4 % adipic acid oleogel sample exhibited the highest oil binding capacity (97.33 ± 2.58 %). - The use of the oleogel in cake resulted in a satisfactory texture profile, as well as acceptable color and organoleptic characteristics.	(Adili et al., 2020)
	Soybean oil was gelled with carnauba wax and adipic acid at concentrations ranging from 0 to 6 %.	Direct dispersion of carnauba wax and adipic acid in soybean oil, followed by heating and cooling to room temperature.	- The addition of adipic acid to carnauba wax-based oleogels led to the formation of new intramolecular or intermolecular hydrogen bonds, improving the thermal behavior and crystallinity of the oleogels. - The formulated food model (cake) with partial substitution of carnauba wax (2 %) and adipic acid (4 %) oleogel as the optimized sample demonstrated an acceptable texture profile, color, and organoleptic characteristics.	(Khiabani et al., 2020)
Breakfast spreads	The wax and glyceryl monostearate were used to gel walnut oil.	The wax and glyceryl monostearate were dispersed in the oil, heated above their melting temperature, and then cooled to refrigeration temperature (0–4 °C).	- The use of candelilla wax resulted in a much firmer spread, with a hardness of 3521 g and a G'LVR of 139,920 Pa, while the monoglyceride-based spread (S-MG) exhibited a hardness of 1136 g and a G'LVR of 89,952 Pa. - The formulated spreads showed shear thinning behavior and increased viscosity with decreasing temperature.	(Pușcaș et al., 2022)
	Food-grade glycerol monostearate gelled corn oil at a concentration of 20 %. Water-in-oleogel emulsions were prepared at water:oleogel weight ratios of 0:100, 45:55, 50:50, and 55:45.	Corn oil oleogel was prepared by directly mixing food-grade glycerol monostearate (20 %) with corn oil. Water-in-oleogel emulsions were produced by mixing water and oleogel at different proportions, followed by homogenization and cooling to room temperature.	- It was found that the emulsion prepared at a 45:55 water:oleogel ratio (45 % replacement of oleogel with water) resulted in chocolate with physical properties similar to the reference sample. - The reduced-fat chocolate spread exhibited comparable sensory scores and acceptability to the full-fat sample.	(Perța-Crișan et al., 2023)
	- Sunflower oil was gelled with shellac wax to form the oleogel. - The oleogel was mixed with water in various proportions to prepare the emulsion oleogels.	- The sunflower oil and shellac wax were directly dispersed to prepare the oleogel. - Water-in-oleogel emulsions were produced by mixing water and oleogel in different proportions, followed by homogenization and cooling to room temperature.	- The emulsions containing 7 % (m/m) shellac oleogels exhibited the strongest behavior, with (G′ & GLVR >30,000 Pa), and showed the highest value of the G′-G″ cross-over. - The whiteness index (Windex) ranged from 58.12 to 78.50.	(Puşcaş & Mureşan, 2022)
Meat products	Ethyl cellulose and adipic acid were used to gel soybean oil at concentrations ranging from 0 to 6 wt%.	Direct dispersion of ethyl cellulose and adipic acid in soybean oil, followed by cooling to ambient temperature.	- Adipic acid improved the thermal behavior and crystallinity of ethyl cellulose-based oleogels. - The 2 % ethyl cellulose/4 % adipic acid oleogel sample demonstrated the highest oil binding capacity (97.33 ± 2.58 %). - The use of the oleogel in meat resulted in a satisfactory texture profile, as well as acceptable color and organoleptic characteristics.	(Adili et al., 2020)
	Soybean oil was gelled with carnauba wax and adipic acid at concentrations ranging from 0 to 6 %.	Carnauba wax and adipic acid were directly dispersed in soybean oil, followed by heating and cooling to room temperature.	- The addition of adipic acid to carnauba wax-based oleogels led to the formation of new intramolecular or intermolecular hydrogen bonds, improving the thermal behavior and crystallinity of the oleogels. - The formulated food model (beef burger), with partial substitution of carnauba wax (2 %) and adipic acid (4 %) oleogel as the optimized sample, showed an acceptable texture profile, color, and organoleptic characteristics.	(Khiabani et al., 2020)
	Beeswax was used as a gelator to form a linseed oil-based oleogel.	Beeswax was directly dispersed in linseed oil, followed by heating and stirring at 80 °C and cooling to room temperature.	- The incorporation of linseed-based oleogel decreased the hardness and adhesiveness of meat pâtés. - A decrease of up to 90 % in the n-6/n-3 (omega-6/omega-3) ratio was observed, implying a better nutritional value of the obtained pâté samples.	(Martins et al., 2020)
	Sunflower oil was gelled with glyceryl monostearate.	5 % (w/w) gelator (glyceryl monostearate, GM) was directly dispersed in the oil phase, then heated at 90 °C and cooled to room temperature.	- The addition of oleogel as a fat substitute made the sausages lighter. - A slight increase in hardness was observed in the sausages with total fat replacement by oleogels.	(Ferro et al., 2021)
	Canola oil was gelled with a 4.5 % chickpea protein dispersion.	Biphasic templating approach followed by freeze-drying.	- The oleogel system, incorporated with plant protein, was developed as a fat substitute, comparable to beef fat in appearance and texture.	(Yen et al., 2023)
Confectionery	6 % carnauba wax was used to gel pumpkin seed oil.	Carnauba wax was directly dispersed in pumpkin seed oil, followed by heating at 90 °C and cooling.	- The oleogel accelerated the second part of the refining process, and its structure was fully recovered in the cream with 100 % efficiency. - A solid-like behavior and good tolerance to deformation were observed for all samples, demonstrating that the oleogel based on pumpkin seed oil and carnauba wax could fully replace cocoa.	(Borriello et al., 2022)
	1.2 g of monoglyceride stearate gelled 10.8 g of palm oil.	Monoglyceride stearate was directly dispersed in palm oil, followed by heating and cooling.	- A positive synergy between oleogel and palm sap sugar improved the heat stability and bloom resistance of chocolate. - The chocolate exhibited the required crystal form, an improved shape retention index, a higher melting enthalpy, and ideal fat bloom resistance, while showing fewer superficial crystals during temperature cycling.	(Chen et al., 2022)
	Sunflower oil-in-water emulsion was gelled with cellulose ether at concentrations of 0.5 %, 1.0 %, 1.5 %, and 2.0 % (w/w).	Emulsion-based oleogel was formed by dispersing cellulose ether at different concentrations in sunflower oil-in-water, followed by drying and shearing.	Partial replacement of cocoa butter (CB) with sunflower-HPMC-based oleogel significantly reduced the hardness of the chocolate.	(Espert et al., 2021)

Oleogels offer a promising avenue for improving the nutritional quality of fried and baked products by replacing traditional solid fats with structured oils. Their ability to maintain semi-solid consistency at room temperature—while being composed largely of unsaturated fats—makes them a strategic alternative for mitigating the health risks associated with trans and saturated fats (Cui et al., 2023). However, the practical application of oleogels in food systems requires more than just a simple substitution; it demands a nuanced understanding of how their microstructure, oil-binding capacity, and thermal stability affect food quality under different processing conditions.

While previous studies have shown that oleogels can reduce oil uptake during frying and preserve the sensory qualities of fried products (Mahmud et al., 2023), this study further confirms their potential as an effective frying medium capable of significantly lowering fat absorption. It demonstrates that adjusting factors influencing oil uptake, combined with the use of different oleogel formulations, can improve texture, decrease oil content, and boost consumer acceptance. Additionally, the findings highlight that oleogels maintain desirable sensory properties and oxidative stability while enhancing the nutritional profile of fried foods. These results reinforce the potential of oleogels to replace conventional oils and support healthier food processing practices.

The extent of these improvements can vary significantly based on the type of oleogelator (e.g., waxes, monoglycerides, ethyl cellulose), oil phase composition, and process parameters (Meng et al., 2018). For instance, recent study explored the oxidative stability of sunflower oil-based oleogels structured with beeswax and its fractions (6 % w/w), addressing the debated role of beeswax in oxidation processes. Beeswax fractions—comprising hydrocarbons, wax esters, free fatty acids, and alcohols—were isolated via preparative flash chromatography and analyzed by TLC and HPLS-ELSD. After 20 days of storage at 35 °C, oxidative indices like peroxide value, TOTOX and anisidine value, and texture (firmness, Young's modulus) were assessed. Unstructured sunflower oil exhibited the highest oxidative stability, while among oleogels, the formulation containing hydrocarbons and monoesters was the most stable. Beeswax-based oleogels had the shortest induction period and a more complex volatile organic compounds (VOCs) profile, including ketones, alcohols, and terpenes, compared to hexanal-dominant profiles in pure oil. Notably, oxidative rate negatively correlated with Young's modulus (r^2^ = 0.9511) and positively with free fatty acid content (r^2^ = 0.8195). These findings suggest that specific beeswax fractions can enhance oxidative resistance in fat-containing products, despite whole beeswax leading to faster oxidation (Sobolev et al., 2022).

The study of Yi et al. (2017) found that increasing wax concentration from 5 % to 15 % in both carnauba wax–canola oil (CWO) and beeswax–grapeseed oil (BWO) oleogels led to higher enthalpy of crystallization and melting, with CWO requiring more enthalpy than BWO. Color differences between oleogels and control oils significantly decreased with higher wax content (*p* < 0.05). BWO exhibited greater oil-binding capacity than CWO. While CWO maintained stable solid-fat content across 10–60 °C, BWO showed a decline. In terms of oxidative stability, CWO demonstrated significantly better performance than control oils at 60 °C and 180 °C (*p* < 0.05), whereas BWO did not improve oxidative stability compared to control oils.

From a nutritional perspective, replacing saturated fats with oleogels rich in polyunsaturated or monounsaturated fatty acids contributes to improved lipid profiles in the diet, potentially lowering the risk of cardiovascular disease (Zetzl et al., 2012). However, this benefit must be weighed against the bioavailability and oxidative stability of these unsaturated oils during storage and high-heat cooking.

The commercial scalability of oleogel-based formulations is another critical area for analysis. While promising at a lab scale, challenges remain in terms of ingredient cost, process adaptability, regulatory approval (in some regions), and consumer perception—particularly regarding unfamiliar ingredients or altered textures.

In conclusion, while oleogels present a transformative opportunity in the design of healthier fried and baked goods, their successful adoption depends on a balanced integration of food engineering, sensory science, and nutritional evaluation. Future research should prioritize comparative studies of oleogel systems across different food categories, evaluate their long-term health implications, and explore synergies with other clean-label and functional ingredients.

### 2.1 Bread and cakes

Bread and cake are culinary creations typically crafted through baking, using a mixture primarily composed of dissolved or dispersed non-fat ingredients such as flour, water, salt, yeast, and milk (El-Sohaimy et al., 2021). Within the bread-making process, fats play multifaceted roles. They enhance gas retention in the dough, increasing its volume and imparting a soft texture. Additionally, fats serve as lubricants, improve aeration, and facilitate heat transfer, contributing to the desirable texture of the final product (Rios et al., 2014).

To reduce the caloric value and fat content of cakes, Pehlivanoglu et al. (2018) formulated cakes using bigels. Their results demonstrated a significant reduction in saturated fatty acid content while achieving higher levels of unsaturated fatty acids in the final product. Similarly, Kim et al. (2017) showed that blending oleogels with shortening (at approximately 25 %) produced cakes with lower saturated fatty acid content while preserving the quality of the final product.

In another study, rice bran wax, beeswax, and candelilla wax were used to formulate sunflower oil oleogels as substitutes for shortening in cakes. The findings indicated that beeswax oleogels were the most promising, yielding cakes with properties comparable to reference samples containing shortening. While rice bran wax and candelilla wax oleogels produced moderately viscous cake batters, their viscosity was lower than that of the control. Substituting shortening with oleogels reduces batter viscosity and shear-thinning tendencies. Incorporating oleogels into cakes significantly lowered saturated fatty acid levels, reducing them from 58 % to as low as 14 %–17 %. Beeswax oleogels resulted in cakes with a softer texture, specific volume comparable to controls, and a porous structure (Oh et al., 2019).

Davidovich-Pinhas et al. (2015) successfully developed oleogels using canola oil and ethyl cellulose, extruded at different concentrations for use in laminated pastries. Recent research has explored ethyl cellulose polymer-structured fats as a substitute for traditional bakery shortenings. Diverse blends of palm stearin and soybean oil were tested, resulting in varying saturated fat levels. Ye et al. (2019) examined ethyl cellulose (EC) oleogels for innovative shortening development. Among different EC variants (EC7, EC100, and EC200), EC100, with an average viscosity of 100 cP, emerged as the most effective organogelator due to its superior hardness and minimal oil migration. The selected shortening, formed with 4 % (wt/wt) EC100 and 1 % (w/w) triglyceride monostearate as an emulsifier, produced breads with excellent specific volume, superior air incorporation, and a consistently soft texture compared to commercial shortening. These findings underscore the potential of ethyl cellulose oleogels to replicate and enhance the functionality of traditional bakery shortenings. By incorporating structured oleogels, it is possible to create breads and cakes with reduced saturated fat content while maintaining desirable quality attributes, offering exciting opportunities for healthier bakery applications.

### 2.2 Cookies and biscuits

Cookies are a popular bakery product enjoyed by people of all age groups. They are typically made using refined wheat flour, which, while rich in fats and carbohydrates, contains relatively low levels of essential nutrients (Ali et al., 2023). In addition, biscuits, and cakes are non-yeasted, laminated baked goods. Their preparation involves interleaving thin layers of dough with layers of fat, a process known as lamination. These fat layers play a pivotal role in defining their characteristic texture (Wickramarachchi et al., 2015). Baked goods typically contain significant amounts of solid fats (15–35 %), with variations in saturated fat content observed across different products: 31.8 % in baking/frying fats, 7.4 % in cakes, 21 % in cookie creams, and 4.1 % in crackers (Schickenberg et al., 2009). More recent studies continue to highlight the high saturated fat levels in commercially baked items, emphasizing the need for reformulation with healthier fat alternatives to improve their nutritional profiles (Das & Das, 2024).

In baking, fat contributes to the microstructure of the dough and stabilizes air cells. In high-fat products, the texture of fat at dough temperature, along with sensory attributes such as flavor, greatly impacts the final product's quality. Reducing or substituting fat with non-fat constituents is more challenging in these products than in others and can significantly affect the properties of the final baked goods. During storage, fat also influences oil migration and oxidative stability.

In Turkey, Mert & Demirkessen (2016a) developed a method for incorporating oleogel and shortening mixtures into cookie formulations using candelilla wax-based oleogels at ratios of 3 % and 6 % to reduce and stabilize saturated fat content. In a related study, Mert & Demirkessen (2016b) assessed the feasibility of using unsaturated oleogels as a shortening replacement in short-dough products. Their findings showed that dough containing oleogel exhibited better consistency and lower hardness compared to the reference. Additionally, the physical structure and properties of cookies were improved when oleogel was included in the formulation, compared to replacing fat with liquid oil.

Hwang et al. (2016) explored the use of vegetable oil oleogels in cookie production, utilizing four types of waxes (sunflower wax, rice bran wax, beeswax, and candelilla wax) and three oils (olive oil, soybean oil, and flaxseed oil). The study found that both the type of wax and the oil significantly influenced dough and oleogel properties, particularly firmness and thermal behavior. Sunflower wax and flaxseed oil produced the firmest oleogels. However, key textural properties of cookies, such as hardness, fracturability, and spread factor, were not significantly affected by the different combinations of waxes and oils.

In another study, Yılmaz & Öğütcü (2015) analyzed cookies formulated with oleogels made from hazelnut oil mixed with sunflower wax or beeswax as a replacement for bakery shortening. They observed that the textural and physical properties of oleogel-containing cookies were comparable to those of commercial bakery cookies. Sensory evaluations using a hedonic test revealed that consumers preferred cookies made with oleogels over commercial bakery cookies.

### 2.3 Meat products

The incorporation of oleogels into meat products, pioneered by various research studies, offers significant potential for the meat industry. This innovation, driven by nutritional and technological considerations, addresses concerns about the health risks associated with trans fats from hardstock sources and the cardiovascular dangers linked to processed meats, which typically contain an average of 35 % saturated fats (de Oliveira et al., 2012). Beyond their nutritional value, fats play an essential role in the flavor, structural integrity, and binding properties that determine the texture and stability of meat products. Given that consumer preferences are closely tied to these quality parameters, it is crucial to preserve the key attributes of traditional meat products while embracing oleogel technology.

To enhance nutritional value and reduce trans and saturated fat content, pork pâté formulations have been developed using oleogels made from ethyl cellulose and sorbitan monostearate or beeswax, combined with olive, linseed, and fish oils. The oleogel emulsions successfully replaced and stabilized pork back fat. Gomez-Estaca et al. (2019) reported that beeswax oleogels are particularly promising for reformulating pork liver pâté due to their ability to improve the product's nutritional profile.

**Fig. 2:**
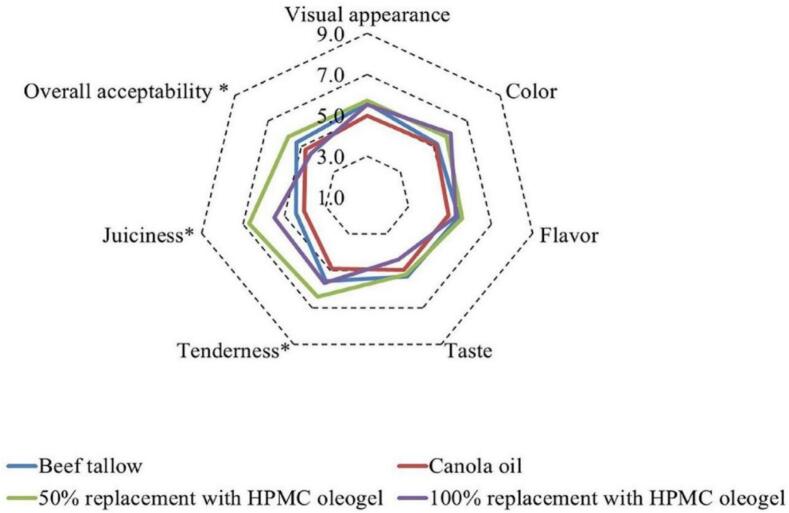
The effect of replacing beef tallow with hydroxypropyl methylcellulose oleogels on the sensory properties of meat patties (Oh et al., 2019).

In another study, Oh et al. (2019) gelled canola oil with hydroxypropyl methylcellulose (HMC) to partially or fully replace beef tallow in meat patties. Textural evaluations revealed that oleogels provided higher values than beef tallow. Patties containing 50 % or 100 % HMC oleogels showed significantly reduced cooking loss. The sample with 50 % substitution achieved the highest sensory acceptability. Moreover, the saturated fatty acid content in patties with HMC oleogels was reduced to 15 %, compared to 42 % in beef tallow patties, without compromising the sensory quality (Fig. 2).

Moghtadaei et al. (2018) investigated the use of beeswax in sesame oil oleogel formation, testing formulations with 5 %, 7.5 %, and 10 % beeswax at room temperature (25 °C) and under cooling conditions (4 °C). Beef flank and shank fats served as reference samples. Although a 50 % reduction in the textural quality of burgers was observed, the oleogel formulations improved color and achieved reductions in fat absorption (1.6 %) and cooking loss (11 %).

In a separate study, an equimolar mixture of γ-oryzanol and β-sitosterol was used to gel linseed oil, which was then incorporated into pork patties at two different proportions. These patties were compared to standard commercial hamburgers. After sous vide cooking, compression tests assessed the hardness and chewiness of the products. Remarkably, pork patties with 25 % oleogel substitution and subcutaneous pork fat displayed similar textural properties and ranked second in sensory evaluations, just behind the reference hamburger. Cost analysis indicated no additional expenses for producing the 25 % oleogel-infused patties (Martins et al., 2019).

**Fig. 3:**
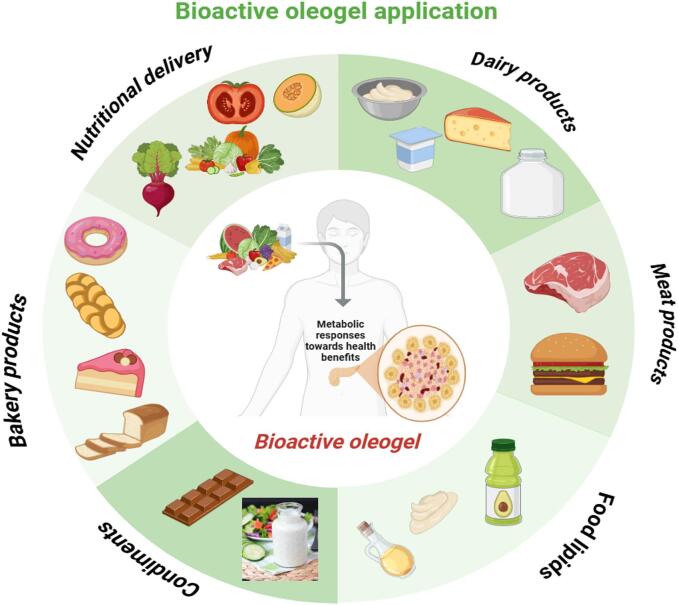
The application of oleogel in various foods products.

Ethylcellulose was found to be an effective organogelator for vegetable oils, forming gels that retained the fatty acid profiles of the oils while exhibiting a solid-like structure suitable for replacing saturated fats in food products. Texture profile analysis and the back extrusion technique were used to evaluate the mechanical properties of oleogels made from canola, soybean, and flaxseed oils, which contained 10 % ethylcellulose and 90 % vegetable oil. The results showed that oils with higher unsaturation levels produced harder gels. Oleogels with different ethylcellulose molecular weights (ranging from 4 to 10 %) demonstrated increased gel strength as the polymer concentration and molecular weight increased. Scanning electron microscopy provided insights into the gel's microstructure. Additionally, frankfurters made with canola oil oleogels were tested as a potential replacement for animal fats, and the cooked frankfurters showed no significant differences in chewiness or hardness compared to the control made with beef fat. This study provided a detailed characterization of ethylcellulose oleogels and suggested their potential for replacing saturated fats in various food products (Zetzl et al., 2012). Fig. 3. Summarizes the application of oleogel in various food products.

## 3 Design of oleogel delivery system enriched with bioactive ingredients

### 3.1 Polysaccharide-based oleogels

Polysaccharides play a critical role in the formulation of oleogels, acting as thickening agents to increase the viscosity of the aqueous phase and improve physical stability, especially when combined with surface-active molecules for stabilizing oil droplets (Espert et al., 2022; Quintana-Martinez et al., 2018). Espert et al. (2022) demonstrated that oleogels prepared with xanthan gum and food-grade structuring agents exhibited high gel strength, elasticity, and excellent oil-binding capacity. Similarly, Huang et al. (2023) found that the type and amount of polysaccharides significantly influenced oleogel characteristics. They reported that the increase of the content of hydroxypropyl methylcellulose in oleogels resulted in a more compact oleogel structure with low droplet size of emulsions, high viscosity and loss modulus.

Hydroxypropyl methylcellulose's influence on the microstructure and rheological properties of oleogels has been well-documented, showing enhanced mechanical strength and oil-binding capacity (Jiang et al., 2021; Jiang & Bai, 2022; Jiang, Li, et al., 2022; Meng et al., 2018). Additionally, the use of sodium alginate and xanthan gum in oleogel formulations induced bridging flocculation, yielding predictable rheological properties (Zambrano & Vilgis, 2023). Oleogels incorporating methylcellulose as an oleogelator with sodium alginate, xanthan gum, or κ-carrageenan showed compact structures, strong oil-binding capacity, and favorable rheological properties. These oleogels also demonstrated high release rates of free fatty acids and curcumin during early in vitro digestion stages (Xu et al., 2023).

Incorporating citrus pectin into camellia oil-based oleogels enhanced oil-binding capacity, gel strength, and thixotropic recovery, with improvements proportional to pectin concentration (Luo et al., 2019). Recent research on emulsion-templated oleogels revealed promising outcomes from protein-polysaccharide complexes. For instance, tamarind seed polysaccharide combined with gelatin resulted in oleogels with excellent oil-holding capacity (oil loss of 3.2 % after extended storage), efficient free fatty acid release during digestion, and strong gel strength (Xie, Ren, et al., 2023).

Moreover, alginate and soy protein conjugate-based oleogels loaded with thymol exhibited robust antimicrobial activity against pathogens like *Staphylococcus aureus* and *Escherichia coli* (Chen & Zhang, 2020). Polysaccharide-lipid complexes also offer potential in oleogel development. For example, double-network oleogels formed by hydroxypropyl methylcellulose and glyceryl monostearate exhibited strong hydrogen bonding, making them suitable fat replacements in cookies and cakes (Jiang, Yu, & Meng, 2022).

### 3.2 Phenolic compounds

Phenolic compounds extracted from natural sources are often incorporated into oleogels to enhance their bioactive content and modify their properties (Butkeviciute et al., 2022; Pan et al., 2020). The addition of phenolic compound-rich extra virgin olive oil to ethyl cellulose oleogels positively influenced the pore size and mechanical properties, including roundness and circularity of the gels (Giacintucci et al., 2018). Oleogels containing tea polyphenols exhibited high antioxidant activity due to the polyphenols' presence, further enhancing their functional properties (Chen et al., 2023; Luo et al., 2019; Shang et al., 2021).

Ferrer-González et al. (2021) reported that incorporating extracts from orange peel, pea pods, and pomegranate peel into oleogels improved their oxidative stability. Similarly, oleogels made from white mustard seeds, rich in p-hydroxybenzoic acid, sinapic acid, quercetin, and epicatechin, showed significant prevention of lipid peroxidation due to their reducing power and chelating activity (Martinovi, Polak, & Abramovi, 2020).

The protective effects of oleogels were further demonstrated by a study on pumpkin seed oil-based oleogels, which preserved phenolic compounds, such as syringic acid and vanillin, during 8 months of storage (Pușcaș, Mureșan, Ranga, et al., 2020). Silva et al. (2022) proposed ethylcellulose and virgin coconut oil-based oleogels, which exhibited high viscoelasticity, thermal stability, good structural integrity, modifiable stiffness, high oil-binding capacity, and antioxidant activity.

In food applications, burgers made with olive leaf extract or olive oil-based oleogels provided antioxidant-enriched end products, improving the oxidative stability of the burgers (Lopes et al., 2022). A key goal in the formulation of oleogels is to improve their resistance to oxidative degradation and antibacterial properties, thus prolonging the shelf life of oil- or fat-based products.

Recently, Günal-Köroğlu et al. (2024) reported that oleogels play a versatile role in encapsulating bioactive compounds like essential oils and phenolics, extending their use to food preservation and delivering lipid-soluble nutrients. Their antibacterial and antioxidant properties, as highlighted in recent studies, underscore their significance in enhancing food quality and safety. Furthermore, oleogel containing phenolic-rich extract displayed cytotoxic effect against colorectal Caco-2 cancerous cells by the down-regulation of anti-apoptotic expression levels of PI3k and COX-2 and up-regulation of iNOS activity (Akl et al., 2025).

**Fig. 4:**
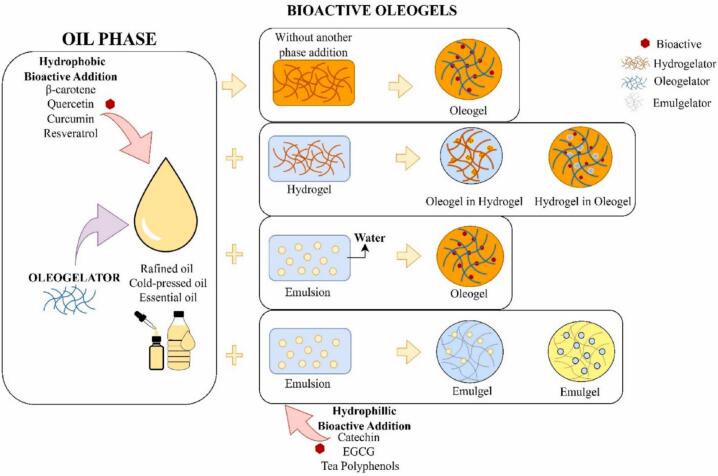
Schematic representation of the process for forming bioactive-loaded oleogels (Günal-Köroğlu et al., 2024).

**Fig. 5:**
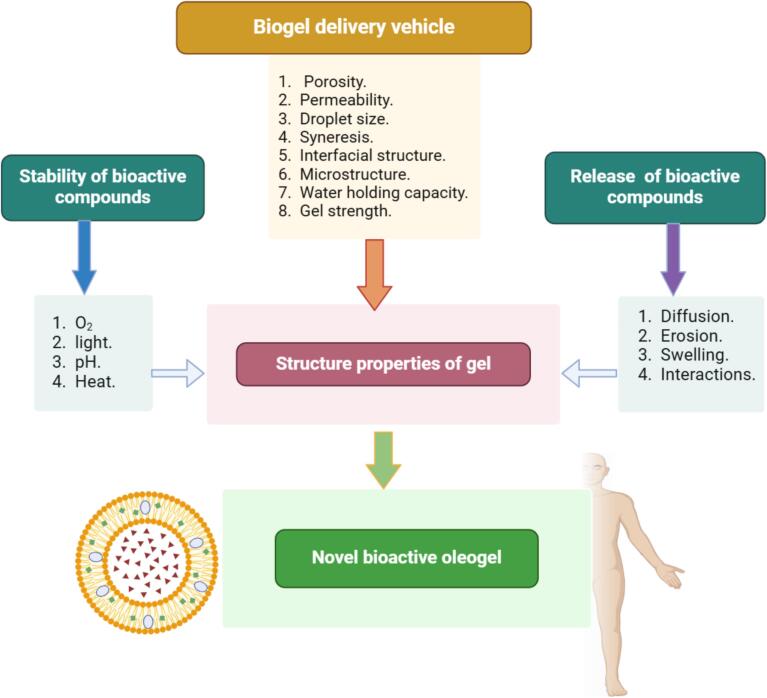
Schematic illustration of the relationship between the structure and functionality of gel-based delivery systems.

In this regards, Fig. 4 illustrates a schematic representation of the process for forming bioactive-loaded oleogels. While Fig. 5 illustrates the relationship between the structure and functionality of gel-based delivery systems (Mao et al., 2020; Okonkwo et al., 2022).

### 3.3 Vitamins

Oleogels have emerged as one of the most promising encapsulants and delivery systems for vitamins. Recent studies have highlighted their potential, showing that oleogels loaded with vitamins exhibit favorable characteristics (Dhal et al., 2023; Wang et al., 2020). Wang et al. (2020) demonstrated that oleogels loaded with vitamin C serve as excellent carriers, with corn-oil-based oleogels displaying the best oxidative stability and physical properties when compared to camellia oil and linseed oil-based oleogels.

Furthermore, oleogels containing β-carotene showed the lowest peroxide values, and muffins made with β-carotene-loaded oleogels exhibited the greatest oxidative stability (Jeong et al., 2021). Policosanol-based oleogels used as carriers for retinyl palmitate (the ester form of vitamin A), successfully protected vitamin A against photodegradation (Tian & Acevedo, 2020). This protective effect is attributed to the physical UV-barrier action, molecular immobilization, and inhibition of free radical-mediated reactions.

### 3.4 Omega fatty acids

Oleogel-based carriers have been effectively designed to retain lipophilic components such as omega fatty acids (Martins et al., 2019). For instance, Lee et al. (2018) developed an oleogel containing β-cyclodextrins, coated with whey protein isolate, in which fish oil was loaded. The study found that 50 wt% of both eicosapentaenoic acid (EPA) and docosahexaenoic acid (DHA) remained after 4 h of UV exposure, and the fish oil odor was reduced. In another study, fish oil was loaded into a beeswax-based oleogel coated with whey protein isolate, which demonstrated strong physical stability during storage and high oxidative stability upon thermal treatment and ultraviolet-C radiation (Lee et al., 2019).

A recent study investigated the potential of beeswax (W-OG) and ethyl cellulose (EC-OG) oleogels—formulated with a health-oriented lipid blend (olive, linseed, and fish oils)—as animal fat replacers in pork liver pâtés. The two oleogels exhibited distinct structural characteristics: W-OG was more rigid and brittle, melting at ∼55 °C, while EC-OG was softer, more flexible, and thermally stable. Both oleogels were used to fully or partially replace pork backfat, resulting in pâtés with enhanced lipid profiles (notably high PUFA/SFA ratios and low n-6/n-3 ratios), meeting EU health claims for omega-3 fatty acids (Gómez-Estaca et al., 2019).

Importantly, the technological (emulsion stability) and physicochemical (texture, color) properties remained comparable to conventional products. However, lipid oxidation increased, especially with higher EC-OG substitution. Sensory analysis revealed no significant changes with beeswax oleogel, while EC-OG had mild negative effects at higher levels, though still rated close to “neutral.” Overall, the oleogels are promising fat replacers for developing healthier meat products with improved nutritional profiles and acceptable sensory qualities (Gómez-Estaca et al., 2019). Furthermore, beeswax oleogels used as pork fat replacers in dry fermented meat formulations improved the fatty acid profile, resulting in a 12-fold decrease in the PUFA n-6/n-3 ratio compared to the control sample, although the sensory attributes of the developed product require further improvement (Pintado & Cofrades, 2020).

Similarly, Martins et al. (2020) reported that the replacement of pork fat with an oleogel rich in linolenic acid for meat pâté formulations resulted in a 90 % reduction in the n-6/n-3 (omega-6/omega-3) ratio, suggesting an increase in omega-3 bioavailability upon digestion. Additionally, studies have shown that the use of oleogels rich in omega fatty acids for food product formulations increases the polyunsaturated fatty acid content while decreasing the saturated fatty acid content (Schubert et al., 2022; Yılmaz & Toksöz, 2022).

### 3.5 Fat-soluble molecules and/or fatty acids encapsulated with oleogels

Encapsulation serves as a transformative strategy that enhances the precision of targeting, controlled release, and bioavailability of bioactive compounds. While bioactive compounds hold immense health potential, their susceptibility to oxidative deterioration and limited chemical stability poses significant challenges. Microencapsulation enhances the delivery of bioactives into food matrices, benefiting minerals, vitamins, probiotics, antioxidants, and fatty acids (Chawda et al., 2017). Through encapsulation, the integrity of bioactive compounds is preserved by fortifying their physicochemical attributes (Mehany et al., 2024), creating functional foods that promote health and exhibit anti-disease activities (Dias et al., 2015).

In the future, it is likely that oleogels will find application as a technique to regulate or decelerate the release of nutraceuticals and pharmaceuticals within food. This is particularly important due to the relatively scarce presence and minimal consumption of fat-soluble molecules such as β-carotene, lycopene, coenzyme Q10, and other bioactive compounds (Ramos-Souza, Fernandes, Mazzo, Perrechil, & De Rosso, 2025). Given their low quantities and bioavailability, it becomes crucial to encapsulate and enhance the delivery system for these active substances (Abd El-Aziz et al., 2024). In this context, utilizing oleogels as a vehicle for transporting these bioactive compounds is emerging as a promising avenue to improve their stability and bioavailability (Pușcaș et al., 2020).

For instance, Li et al. (2021) proposed an ultrasound-assisted gelation method for β-carotene-enriched oleogels based on candelilla wax and nut oils, applying in vitro digestion analysis. The authors observed that high-intensity ultrasound (95 W, 10 s) reduced the liberation of β-carotene during intestinal digestion. They concluded that high-intensity ultrasound may enhance the functional attributes of wax-nut oil oleogels and their ability to encapsulate β-carotene.

In another study, Chinnabutr and Chamchong (2022) employed ethyl cellulose (0–7 %) and beeswax as oleogelators to trap bioactive compounds from gac (*Momordica cochinchinensis*) oil. After 3 months of storage at room temperature, they found that oleogels prepared with 5 % ethyl cellulose significantly improved the contents of lycopene (11.61 mg/100 g), β-carotene (3.02 mg/100 g), and antioxidant activity.

Zhu et al. (2021) developed bigels incorporating lycopene by blending glycerol monostearate-beeswax-based oleogels with high-acyl gellan gum hydrogels in varying ratios. During in vitro simulated gastrointestinal digestion, lycopene release ranged from 60 % to 80 %. The study found that increasing the proportion of oleogel within the bigel matrix reduced the release rate of lycopene, indicating that a higher oleogel content may enhance the delivery efficiency of fat-soluble nutraceuticals through the gastrointestinal tract.

In a similar study, Zheng et al. (2020) employed κ-carrageenan hydrogel and monoglyceride oleogels to entrap β-carotene. They found that bigels formulated with 50 % oleogel enhanced the light and thermal stability of β-carotene, with about 70 % retention after 8 h under UV light exposure in bigels prepared with 75 % oleogels. Moreover, Dhulipalla et al. (2023) incorporated lycopene into oleogels prepared with coconut oil and stearic acid. The study observed that samples formulated with a combination of 50 % lycopene and stearic acid exhibited a firm texture and improved stability.

Numerous studies have also focused on using oleogels to improve the stability and bioavailability of coenzyme Q10 (CoQ10), docosahexaenoic acid (DHA), and eicosapentaenoic acid (EPA) (Zulfakar et al., 2018; Wang et al., 2020). Masotta et al. (2019) demonstrated that high-dose CoQ10-loaded ethyl cellulose-based oleogels enhanced its retention for 12 months, showing high oxidative stability for oral therapeutic administration.

Polyunsaturated fatty acids (PUFAs), such as eicosapentaenoic acid (EPA) and docosahexaenoic acid (DHA) within fish oil and other edible oils, are particularly vulnerable to oxidation due to their elevated unsaturation levels. Previous research has shown that converting oils into structured lipids (SL) can further reduce oxidative stability (Jennings & Akoh, 2001; Willett & Akoh, 2018). One potential way to enhance resistance to oxidation in SL is through the creation of oleogels. Wang et al. (2022) proposed a technique to limit the oxidation of polyphenols and omega-3 polyunsaturated fatty acids (n-3 FAs), particularly DHA, EPA, and conjugated linoleic acid, by embedding them in oleogels formulated with algal oil and polyphenol-enriched whey protein microgel particles as gelling constituents. Their findings demonstrated that protein oleogels significantly reduce the challenges associated with formulating n-3 FAs and improve their oxidative stability, sensory attributes, and textural properties, offering integrated health benefits.

### 3.6 Oleogels with protein and amino acids

In food formulations, proteins play a vital role in enhancing the texture and rheological properties of a wide range of products. Their nutritional value, exceptional water solubility across various pH levels, and unique techno-functional attributes contribute to their widespread application (Elsohaimy et al., 2015). Under specific physico-chemical conditions, proteins can form structured gel networks in aqueous environments, driven by their amino acid sequence and secondary structure (Schmitt et al., 2019). The process of protein gelation stems from a dynamic interplay between attractive and repulsive interactions, ultimately leading to aggregation. This phenomenon is orchestrated through various pathways, including adjustments in temperature, addition of salts, cooling processes, and pH modulation around the protein's isoelectric point (Plazzotta et al., 2020; Nicolai, 2019).

To generate protein oleogels, two categories of proteins—globular and fibrous—are frequently utilized. Globular proteins, such as egg albumin, β-lactoglobulin, soy protein, and whey protein isolate, can form gels primarily through intermolecular interactions facilitated by heat-induced denaturation. Elevated temperatures prompt the energetic motion of peptide chains within globular proteins, causing amino acids to interact and bind with other protein molecules, fostering aggregation that ultimately leads to the formation of a three-dimensional network (Nicolai, 2019). A significant advantage of employing thermosetting protein aggregates in gel formation is their adaptability across various globular protein types.

On the other hand, fibrous proteins like gelatin adopt a flexible polyelectrolyte chain configuration in aqueous solutions at elevated temperatures. Upon cooling, these proteins engage in hydrogen bonding interactions, which induce aggregation and subsequent gelation (Djabourov et al., 1988; Sintang et al., 2021).

Recently, there has been an increased focus on oleogels formulated from globular proteins. These studies encompass diverse sources, including soy protein (Naji-Tabasi et al., 2020), whey protein (Plazzotta S et al., 2020; Sintang et al., 2021), and egg protein (Jaberi et al., 2020). In the study by Meissner et al. (2020), a protein oleogel system was designed using a solvent-exchange approach. Their findings revealed that whey protein facilitated the gelation of high oleic safflower oil. Whey protein isolate (BiPro) was obtained from Agropur Inc. (Appleton, USA) containing 97.7 % protein and 75 % beta-lactoglobulin in dry matter. Notably, this protein-mediated gelation offered additional benefits as an active antioxidative agent, mitigating the degradation of lipid oxidation products such as aldehydes in the prepared oleogels.

In the study of Naji-Tabasi et al. (2020), oleogels were prepared using a Pickering emulsion-templated method with isolated soy protein (ISP) and basil seed gum (BSG) as stabilizing agents. ISP-BSG particles were formulated at different mass ratios—1:0 (1S:0B), 1:1 (1S:1B), 2:1 (2S:1B), and 3:1 (3S:1B)—to evaluate their effects on oil retention, mechanical strength, network compactness, thermal stability, and consistency. The 2S:1B ratio demonstrated superior performance, producing a more stable oleogel with higher mechanical strength, a more compact network, and better oil bonding capacity. Viscoelastic testing confirmed gel formation in the oleogel system. These formulations were then applied to produce creams with 5 %, 10 %, and 15 % fat reductions, with the 2S:1B formula at 5 % fat reduction receiving the highest overall sensory acceptance, showing no significant difference from the full-fat control cream.

**Fig. 6:**
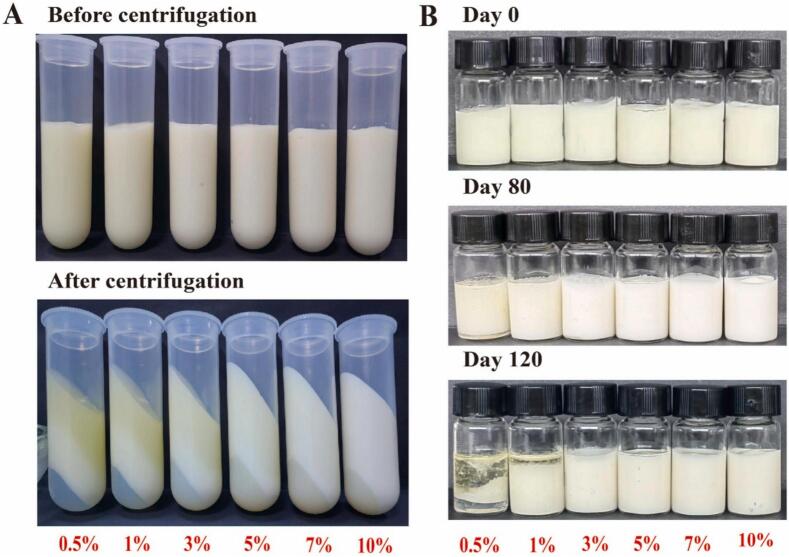
Stability of Pickering emulsion at different protein concentrations. (**A**) Centrifugal stability. (B) Storage stability. (Sun et al., 2024).

Recently, Sun et al. (2024) prepared a Pickering emulsion (PE) stabilized by whey protein isolate/soybean protein isolate (WPI/SPI) composite particles and encapsulation of milk-derived peptide FDRPFL. WPI and SPI (purity ≥95 %) were purchased from Linyi Shansong Biological Products Co., Ltd. Additionally, the stability of PEs was evaluated based on centrifugation and storage stability (Fig. 6). Upon centrifugation, the samples exhibited distinct layering: the top layer consisted of the oil fraction, the middle layer was the emulsion, and the bottom layer represented the water phase, as shown in the appearance diagram. Moreover, the gels, optimized with sodium alginate (SA), showed a dense network structure, high encapsulation efficiency (72.8 %) for peptide FDRPFL, and improved peptide bioaccessibility. These findings highlight the potential of PEGs for advanced food and nutraceutical applications.

**Fig. 7:**
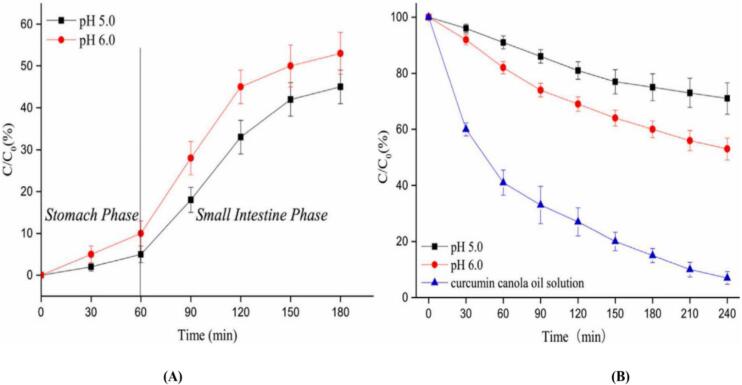
**(A)** Release of curcumin from Pickering emulsion gels stabilized with WPI particles prepared at pH 6.0 or 5.0 under simulated gastric or intestinal digestion conditions. **(B)** Degradation rates of curcumin under light irradiation in Pickering emulsion gels stabilized with WPI particles prepared at pH 6.0 or 5.0, compared to a canola oil solution. *C* and *C*_*0*_ represent the curcumin concentration at a specific time and at time zero, respectively (Lv et al., 2020).

Milk proteins, commonly used as a protein source, are being explored for their potential to stabilize Pickering emulsion gels and offer health benefits. WPI powder (purity >95 %) was supplied by Davisco Foods International Inc. (MN, USA). When whey protein isolate (WPI) was subjected to high hydrostatic pressure and mechanical homogenization (100 MPa) near its isoelectric point (pH 5.0), it formed Pickering materials (particles) with neutral wettability (Lv et al., 2020). The most significant self-aggregation of WPI occurred at pH 5.0, as the electrostatic repulsion between WPI molecules decreased, enhancing their interfacial activity (Lv et al., 2020). Pickering emulsion gels containing 2.0 % of these materials, a 50 % oil phase, and 0.75 % curcumin showed high stability and curcumin encapsulation efficiency. The highest encapsulation efficiency of curcumin, around 90 %, occurred at pH 5.0. Curcumin in these gels exhibited better release under simulated intestinal conditions than under gastric conditions (Fig. 7 A) and was resistant to photodegradation (Fig. 7 B) and After 240 min of light exposure (0.35 W/m^2^, 35 °C), about 70 % of curcumin remained stable in the Pickering emulsion gels at pH 5.0, compared to over 53 % at pH 6.0. The protection of curcumin by these gels was attributed to their network structure, which enhances stability and prevents the diffusion of pro-oxidants and free radicals, thus safeguarding the curcumin. During an in vitro digestion test, curcumin release was minimal in the stomach and approximately 40 % in the intestine. The emulsion gels were resistant to pepsin; however, under intestinal conditions, the combined effects of pH, bile salts, and proteolytic enzymes led to the breakdown of the emulsion gel structure, promoting curcumin release.

**Fig. 8:**
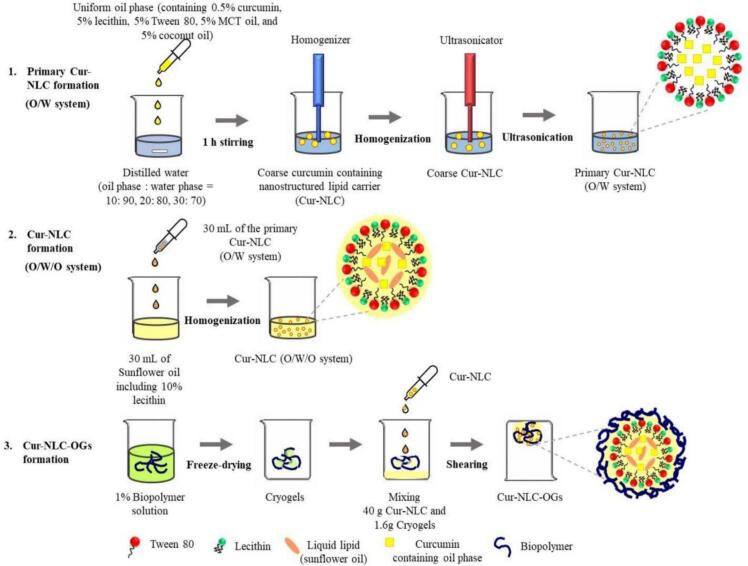
Schematic diagrams illustrating the preparation of curcumin-nanostructured lipid carrier-oleogels (Cur-NLC-OGs) through three steps: **(1)** primary Cur-NLC (O/W system) formation, **(2)** Cur-NLC (O/W/O system) formation, and **(3)** Cur-NLC-OGs formation (Zhang, Chuesiang, et al., 2022).

Additionally, curcumin-nanostructured lipid carrier-loaded oleogels (Cur-NLC-OGs) were developed using biopolymer cryogels to improve curcumin's solubility and stability (Zhang, Chuesiang, et al., 2022). The Cur-NLC-OGs exhibited higher encapsulation efficiency, slower curcumin release under acidic conditions, and enhanced control of lipid digestion, particularly with CMC-based cryogels. These findings emphasize their potential for functional food applications. The curcumin-loaded NLC (Cur-NLC) was prepared through a two-step emulsification process (Fig. 8).

### 3.7 Natural essential oils

Essential oils (EOs) are mixtures of lipophilic and volatile compounds in the form of odorous liquids derived from plants. Notably, compounds such as terpenoids, sesquiterpenoids, and other volatile constituents are used as natural ingredients in the treatment of various conditions, including inflammation, cardiovascular diseases, diabetes, and metabolic syndrome (Tyagi et al., 2020). EOs also possess antibacterial properties, as demonstrated by several studies (He et al., 2020; Miladinović, et al., 2021). However, the hydrophobic nature, strong flavor, and pungency of EOs limit their broader applicability. To overcome these limitations, incorporating EOs into suitable delivery systems compatible with food applications is essential. Nanoemulsions and nanoparticles encapsulating EOs in water have been widely explored (Prakash et al., 2018; Hadidi et al., 2020), but the use of EOs as oleogels for food applications remains underexplored and warrants further investigation.

For example, Kasparaviciene et al. (2018) prepared an oleogel with thyme essential oil, known for its antimicrobial activity, under optimal formulation conditions and evaluated the effect of ingredients on the textural structure. The study found that oleogel containing 0.05 % thyme essential oil significantly inhibited *Candida albicans*. In a recent study by Necla Ozdemir & Zeynep (2022), black cumin oil combined with natural waxes (beeswax, sunflower wax, and carnauba wax) was used to produce oleogels. The black cumin essential oil oleogel with carnauba wax showed the lowest activation energy (354.82 kJ/mol), indicating greater resistance to thermal degradation. All oleogels displayed weak gel properties (tan δ > 0.1), but the black cumin oil oleogel effectively protected the oil from oxidation over an eight-week storage period.

Lertsatitthanakorn et al. (2008) studied the antibacterial activity of citronella oil oleogel against *Propionibacterium acnes* and its physical and chemical stability. The research demonstrated that solid lipid particles encapsulated in citronella oil extended the oil's release, with the oleogel retaining anti-*P. acnes* activity even after 120 days at 40 °C, unlike unencapsulated citronella oil, which lost effectiveness by day 45. The solid lipid wall protected the oil's constituents from volatility, enhancing the oleogel's stability. These findings suggest that formulations of topical anti-acne treatments incorporating solid lipid particles loaded with citronella oil oleogels could offer promising alternatives for acne therapy.

In a recent study by Uronnachi et al. (2022), oleogels formulated from rosemary and cedarwood essential oils were tested for their hair growth-enhancing effects. The oleogels were prepared using beeswax as the organogelator, at concentrations of 10 % for singular oils or 5 % each for combined oil preparations. Differential scanning calorimetry (DSC) thermograms of the oleogels exhibited varying degrees of amorphicity. The rosemary oil oleogel showed higher oil binding capacity (91 %) compared to cedarwood oil (81 %) and enhanced spreadability. In terms of hair growth, the rosemary oil oleogel (10 %) showed similar effects to the positive control (2 % Minoxidil) over a six-week period.

### 3.8 Bioactive compounds from food wastes

Bioactive substances, which are derived from plants, can be classified into several groups, such as phytosterols, terpenes, polyphenols (flavonoids, phenolic acids, stilbenes), and alkaloids. While phytochemicals are considered non-essential nutrients, they play a vital role in human health due to their significant antioxidant, anti-inflammatory, and antimicrobial properties (Mehany et al., 2021). These compounds can influence cell differentiation, promote apoptosis in cancer cells, and mitigate cell proliferation (Thakur & Belwal, 2023). A large volume of waste and by-products is generated from the agri-food sector, containing substantial amounts of phytochemicals. These compounds can be harnessed in crosslinking structures for the engineering of emulsions, oleogels, or oleofoams. Moreover, food waste from fruit peels, pomace, seeds, and related by-products is rich in phytonutrients, which are ideal for developing novel food products with nutraceutical properties.

However, the use of bioactive compounds in their native forms is often limited due to low stability and bioavailability. These challenges can be overcome by incorporating bioactives into colloidal structures, such as oleogels (Liu et al., 2023). For example, phenolic acids (e.g., tannic and sinapic acids), phytosterols (e.g., β-sitosterol, γ-oryzanol), and terpenes (e.g., Quillaja saponin, β-carotene), extracted from food wastes, have proven effective as cross-linking agents in the formulation of oleogels and oleofoams, showing promising results in various food systems. The application of phenolic acids as cross-linkers enhances the thermo-mechanical properties of colloidal systems and improves their resistance to various stress conditions.

In the study by Qiu et al. (2018), nanocomplexes formed through interfacial adsorption of tannic acid, proteins, and polysaccharides were used to create a robust oleogel. This gel exhibited excellent thixotropic recovery and rehydration capabilities, ensuring stability. Truong et al. (2019) demonstrated that the use of native phytosterols (a mixture of sterols and stanols) for co-crystallization with acylglycerols led to the formation of stable oleofoams. Additionally, leveraging β-sitosterol and medium-long chain diacylglycerol (MLCD) for Pickering stabilization and network formation resulted in oleofoams with regulated volatile release (Qiu et al., 2021). In a study by Chen & Yang (2019), a self-assembly mechanism using Quillaja saponin as the exclusive gelator led to the formation of an interfacial film with fibrous networks. This oleogel exhibited adjustable rheological and textural properties, along with thixotropic recovery. These findings highlight the potential application of Quillaja saponin as a singular phytochemical gelator in oleogel formation. Table 2 provides a summary of bioactive compound delivery in food and medical oleogel systems, highlighting key findings from recent studies.

**Table 2:** A summary of the delivery of some bioactive compounds in food and medical oleogel systems and the main findings.

**Bioactive** **compound**	**Health benefits**	**Oil**	**Gelator**	**Concentration (%) of gelator**	**Type of** **structure**	**Main findings**	**Reference**
Isolariciresinol, vanillin, caffeic and syringic acids	Antioxidant activity	Pumpkin seed oil	Beewax, sunflower wax, and rice bran wax	3 % of beeswax, 1 % of sunflower wax, and 1 % of rice bran wax	Oleogel	- During the storage at room temperature, it was concluded that oleogelation technique might show potential protection of specific phenolic compounds such as syringic acid and vanillin after 8 months of storage. - Oleogelation exhibited antioxidant effects.	(Pușcaș et al., 2020)
Lidocaine	Analgesic and antiarrhythmic effects	Olive oil	TWEEN80, and SPAN80	15–25 g	Oleogel	This study developed olive oil-based oleogels enriched with lidocaine for potential medical use, particularly in pain relief. It compared the effects of two surfactants—TWEEN 80 and SPAN 80—on oleogel properties. SPAN 80-stabilized oleogels demonstrated superior stability and mechanical strength, making them more suitable for pharmaceutical applications. Structural, rheological, sorption, and wettability analyses confirmed that surfactant type and concentration significantly influence oleogel performance and stability, with SPAN 80 offering the most promising results.	(Kudłacik-Kramarczyk et al., 2024).
Rutin, hyperoside, isoquercitrin, avicularin, quercitrin, quercetin, procyanidin B2, (+)-catechin, and (−)- Epicatechin, phlorizin, and chlorogenic acid	Antioxidant activity	Olive oil	Poloxamer	18 %	Oleogel	The oleogel-released complex of phenolic compounds penetrating into the epidermis showed the strongest DPPH free radical scavenging activity.	(Butkeviciute et al., 2022)
Polyphenols and curcumin	Antioxidant effect	Soybean oil	Glyceryl monostearate	15 g	Oleogel	This study successfully developed antioxidant-fortified oleogels by encapsulating hydrophilic tea polyphenols (TP) in glyceryl monostearate (GMS), enabling their uniform dispersion in lipid-based systems. Compared to lipophilic curcumin, TP showed a stronger antioxidant effect, significantly enhancing oleogel oxidative stability. A synergistic interaction between TP and curcumin was also observed. This approach offers an effective strategy for incorporating hydrophilic bioactive compounds into oleogels, expanding their potential in functional lipid-based products.	(Chen et al., 2023)
Polyphenols	Antioxidant effect	Soybean oil	-Candelilla wax -Ethyl cellulose, alpha-cellulose and Avicel RC-591	3 % wax and 11 % cellulose	Oleogel	Oleogels elaborated with celluloses exhibited better oxidative stability.	(Ferrer-González et al., 2021)
Polyphenols and tocopherols	Oxidative stability	Olive oil	Pork skin powder	Pig skin, deionized water, and olive oil at a 1:3:1 ratio	Oleogel	- The antioxidant content in the virgin olive oil used in the oleogels efficiently prevented the oxidation of burgers for 45 days under normal packaging and for more than 90 days of storage under vacuum. - Good oxidative stability of all burgers during storage at 4 °C and at −20 °C for at least one and a half months.	(Lopes et al., 2022)
Cinnamaldehyde	Antimicrobial activity	Cinnamon oil	Ethyl cellulose	10 %	Oleogel emulsion	This study demonstrated that ethyl cellulose (EC) viscosity plays a key role in structuring cinnamon essential oil (CEO) into oleogels with enhanced stability and antibacterial activity. Higher EC viscosity resulted in more compact oleogel networks and larger emulsion particle sizes. Among the tested variants, the 45 cP CEO-EC emulsion showed the strongest antimicrobial effect against *E. coli* O157:H7 and *S. aureus*, both in model systems and sausage applications. These findings highlight the potential of CEO-EC oleogels as natural antibacterial agents for meat preservation, especially in traditional Chinese ready-to-eat sausages lacking sealed packaging.	(Zhang et al., 2019)
Tymol and carvacrol	Antifungal activity	Olive oil	Aerosil	n.i	Oleogel emulsion	- Oleogel with thyme essential oil affects the *Candida albicans* microorganism when the thyme essential oil concentration is 0.05 % in the oleogel mixture.	(Kasparaviciene et al., 2018)
Surfactin as an antibacterial and emulsifier agent	Anti-infammatory activity	Grapeseed oil	Gamma polyglutamic acid	0.4 g	Surfactin-oleogel	- Surfactin-oleogel effectively reduced *P. acnes*-induced epidermal swelling and erythema. - Surfactin-oleogel decreased epidermal thickness to 48.52 % compared to the model control group. - The *P. acnes* colony in the epidermis was reduced by 1 log CFU/mL following surfactin-oleogel treatment.	(Shan et al., 2022)
Beta-carotene	Antioxidant activity	Canola oil	Ethylcellulose	10 %	Oleogel	The structural and mechanical strength of the elaborated oleogel influenced lipolysis and transfer. Ethyl cellulose oleogelation protected β-carotene from degradation under accelerated storage conditions compared to a heated canola oil control.	O’Sullivan. et al. (2023)
Terpenoids	Hair growth potential	Cedar oil and rosemary	Propylene glycol	26 g olive oil and 8 g beewax	Oleogel	Oil binding capacity values were higher with rosemary oil than with cedarwood oil, ranging from 91 % (for the oleogel containing 10 % rosemary oil) to 81 % for the bland oleogel (without essential oil).	Uronnachi et al. (2022)
Terpenoids	Antibacterial activity	Citronella oil	Aerosil	6 g	Oleogel	The anti-*P. acnes* activity of the oleogel with citronella oil-loaded solid lipid particles remained active, whereas the oleogel without citronella oil became inactive by day 45. Furthermore, the solid lipid barrier protected against the volatility of the oil constituents in the oleogel and prolonged the release of citronella oil.	(Lertsatitthanakorn et al., 2008)

n.i = not indicated.

## 4 Bioavailability of oleogels delivery system for bioactive compounds

The low bioavailability and rapid degradation of bioactive compounds under physiological conditions present significant challenges for their clinical and nutritional use. Addressing these issues requires the design, development, and optimization of systems that can encapsulate, protect, transport, and release these bioactive compounds (Ehrenhaus Masotta et al., 2020; Vellido-Pérez et al., 2019). Oleogels have emerged as an effective encapsulation and delivery system, offering protection to bioactive compounds from degradation in the digestive tract (O'Sullivan et al., 2016; Palla et al., 2022; Vellido-Pérez et al., 2019).

Vellido-Pérez et al. (2019) optimized oleogel formulations, this study focused on optimizing an oleogel formulation designed to stabilize and transport curcumin while protecting the lipid phase (mainly fish oil concentrate) from oxidation. Using a Box-Behnken Design, the researchers investigated the effects of curcumin concentration, structurant concentration, and manufacturing temperature on the oleogel's oxidation degree and curcumin stability. The optimal oleogel formulation was determined for two storage temperatures:

• For 23 °C storage: 0.150 wt% curcumin, 4.461 wt% structurant, and a manufacturing temperature of 64.63 °C.
• For 40 °C storage: 0.150 wt% curcumin, 7.000 wt% structurant, and 62.82 °C.

The results suggest that oleogels are effective for transporting and protecting bioactive compounds like curcumin, highlighting their potential in preventing lipid oxidation and enhancing curcumin's chemical stability.

After two hours of intestinal digestion, the release rate of free fatty acids loaded in oleogel-based emulsions was found to be between 55.04 % and 66.37 % (Dong et al., 2020). The characteristics of oleogels, such as microstructure and gel strength, were shown to significantly influence the release rate of free fatty acids (Dong et al., 2020). Additionally, curcumin-loaded hydroxypropyl methylcellulose oleogels promoted the release of free fatty acids and curcumin during in vitro lipid digestion, although increasing the content of hydroxypropyl methylcellulose might increase viscosity (Chuesiang et al., 2022). In this study, the release rates were estimated to be 25 % for curcumin and 35 % for free fatty acids after in vitro digestion (Chuesiang et al., 2022). The increase in viscosity in oleogels can hinder the bioavailability of bioactive compounds.

Zhang et al. (2025) reported on the engineering of the crystal network in supramolecular oleogels through kinetic regulation to improve lutein bioaccessibility. This study demonstrated that controlling the cooling temperature and aging period of supramolecular oleogels affects their crystal network, which in turn influences their physicochemical properties. Oleogels with denser crystal networks exhibited higher lutein bioaccessibility, likely due to better protection against gastric acid degradation and enhanced micellar capacity. These findings highlight how regulating kinetic factors can optimize oleogels for the delivery of lipid-soluble bioactive compounds.

**Fig. 9:**
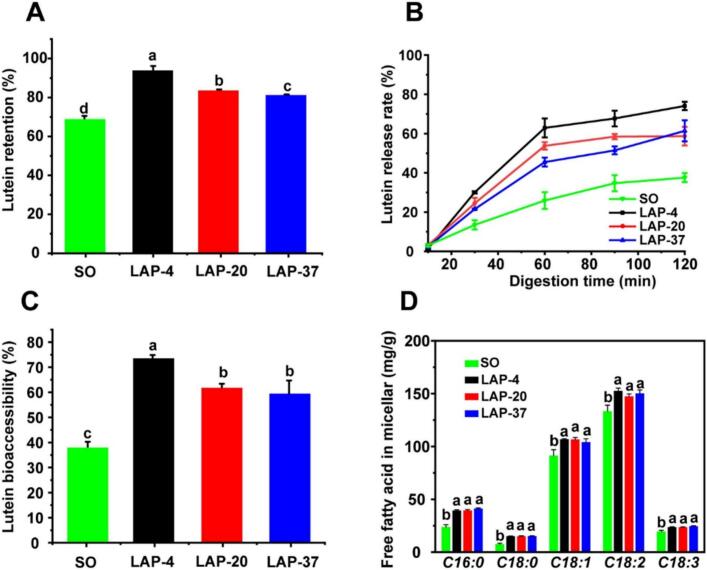
Retention rate of lutein **(A)** after in vitro gastric digestion, lutein release rate **(B)** during in vitro intestinal digestion, and lutein bioaccessibility **(C)** of SO and supramolecular oleogels. Free fatty acid composition (mg/100 g) of the micellar fraction **(D)** in the intestinal-digested SO and oleogels. Different letters indicate significant differences (*p* < 0.05). Data are presented as the mean ± SD (*n* = 3). (Zhang et al., 2025).

The release properties of lutein are shown in Fig. 9. Panel  A in Fig. 9 illustrates the lutein retention rate after in vitro gastric digestion, with the highest retention observed in LAP-4. This is likely due to the formation of a compact crystal network and high mechanical strength in the supramolecular oleogels, which resisted mechanical disruption during digestion, preserving the integrity of the crystal network and reducing lutein's contact with gastric fluid (Dent et al., 2022).

For example, Qu et al. (2022) found that oleogels are effective carriers for resveratrol; however, they may reduce the bioavailability of resveratrol when loaded into oleogels. Furthermore, the composition of oleogels significantly influences their ability to act as carriers and the bioavailability of bioactive compounds after in vitro digestion (Qiu et al., 2023). In another study, the bioaccessibility of β-carotene in oleogel-in-water Pickering emulsions (68.17 %) was higher compared to conventional oil-in-water Pickering emulsions (53.15 %), suggesting that oleogels may be more suitable for delivering hydrophobic and indigestible bioactive compounds (Qi et al., 2020). El-Dahmy et al. (2023) reported that oleogels facilitated the bioavailability of olmesartan medoxomil by 2.5 to 4.5 times more than standard gels and oral market tablets, respectively. Table 3 presents data on the bioavailability of various bioactive compounds delivered through food oleogel systems.

**Table 3:** Bioavailability of bioactive compounds in food oleogels systems.

**Compounds incorporated in oleogels**	**Analyzed oleogels**	**The main findings on bioavailability**	**Reference**
Free fatty acids	10 wt% phytosterols (γ-oryzanol and β-sitosterol) were directly dispersed in 90 wt% sunflower oil to form the oleogel.	This study investigated how self-assembled microstructures in phytosterol oleogels (containing γ-oryzanol and β-sitosterol) affect lipid digestibility. The results showed that oleogels were harder to emulsify than oils, with emulsification efficiency linked to gel strength and crystal structure. Oleogels also led to less lipid droplet coalescence and smaller particle sizes during digestion. The release of free fatty acids (FFAs) was slower, suggesting that oleogels control lipid digestion by limiting lipase-lipid interactions. These findings offer insights into designing oleogels to control lipid digestibility and the bioavailability of bioactive compounds.	(Dong et al., 2020)
Olmesartan medoxomil	Lavender oil was mixed with Tween 20 and Aerosil 200 to obtain the oleogel.	- The oleogel increased olmesartan medoxomil release by 4.21 and 4.97 times compared to the drug suspension and gel, respectively. - The oleogel formulation enhanced olmesartan medoxomil permeation by 5.62 and 7.23 times compared to the drug suspension and gel, respectively. - The pH of the semisolid formulations can affect the solubility and stability of the loaded drug and can influence its skin irritation potential. Thus, oleogel formulations should have pH values within the physiologically accepted range (4.5–6.5) to be safe and non-irritant. The pH values of all the prepared oleogel formulations were within the required range, where they were found to be from 4.91 ± 0.27 to 6.20 ± 0.31.	(El-Dahmy et al., 2023)
Resveratrol	Different multi-component oleogels, including carnauba wax oleogels, β-sitosterol/lecithin oleogels, and ethyl cellulose oleogels, were prepared by the direct method using either peanut oil or soybean oil.	- The bioavailability of resveratrol loaded in oleogels ranged from 45 % to 71.22 %. - The network structure of the different oleogels and the molecular properties of the oleogelators led to variations in the in vitro digestion of the multi-component oleogels. - Differences in oleogel processing conditions altered the degree of lipolysis and the bioavailability of resveratrol within them. Overall, the various gelation mechanisms used in oleogel formation exhibited significant differences in their susceptibility to digestive lipolysis (p < 0.05). Therefore, depending on the intended application, an appropriate oleogel system can be selected as a carrier for specific lipophilic bioactive substances (LBAS). In this study, the RE-loaded oleogel formulation showing the highest extent of lipolysis was the binary soybean oil (SO)– β-sitosterol/lecithin oleogels (S/Los) system (39.74 ± 2.14 %), while the lowest was observed in the quaternary PO–ECO system (17.34 ± 0.37 %).	(Qiu et al., 2023)
Resveratrol	Soybean oil and peanut oil were gelled with β-sitosterol and lecithin.	- Oleogels made from soybean and peanut oils, loaded with resveratrol (R) and blueberry extract, was developed as an alternatives to trans and saturated fats in food. The oleogels maintained their structure, improving mouthfeel and hardness, with peanut oil-based oleogels showing better physical stability over time than soybean oil-based ones. Although the oleogels effectively carried resveratrol, they reduced its bioavailability. The oleogels also demonstrated good thermal stability and strength, making them a promising option for stable, nutritious lipid alternatives. - In addition, in vitro studies of bioavailability reported that oleogels could be used as carriers for lipid substances (up to 57.73 %).	(Qu et al., 2022)
Curcumin	Dispersion of hydroxypropyl methylcellulose in sunflower oil	- High-viscosity grade HPMC maintained the strength and durability of oleogels against mechanical and enzymatic breakdown. - The remaining fractions of curcumin absorbed in the GI tract of rats were primarily influenced by the form of curcumin in the oleogels.	(Chuesiang et al., 2022)
Lycopene	Oleogel was prepared by dissolving glycerol monostearate (1 %, w/w) and beeswax (BW) (1 %, w/w) in soybean oil under agitation at 85 °C until complete solubilization.	The total release percentages varied from 60 % to 80 %, and a higher content of oleogel within bigels slowed down the release of lycopene, suggesting that a higher proportion of oleogel was beneficial for the delivery of fat-soluble nutraceuticals.	(Zhu et al., 2021)
Curcumin	Corn oil-based oleogels were prepared with carnauba wax, behenyl alcohol, and a CW-BA mixture at a 1:4 ratio.	This study explored the preparation of lactoferrin particles (TG-LF) using transglutaminase-induced crosslinking, with optimal conditions at pH 8, 100 U/g TG concentration, 50 °C, and 2 h of crosslinking time. TG-LF particles had a contact angle of 79°. The in vitro digestion study showed that the CW oleogel-based Pickering emulsion had the highest lipolysis rate and curcumin bioaccessibility. The findings suggest that TG-LF particle-stabilized oleogel-based Pickering emulsions are effective for curcumin delivery, offering a novel approach for developing Pickering emulsifiers.	(Xia et al., 2022)

## 5 The opportunities of utilizing oleogel delivery systems for bioactive compounds

I. In this decade, oleogels have emerged as a promising encapsulation and delivery system for bioactive compounds. However, the application of oleogels as a delivery system presents some drawbacks that limit their efficiency. Despite the wide variety of formulated oleogels expanding their application spectrum, these variations can impact the physicochemical and molecular structures of oleogels (Günal-Köroğlu et al., 2024; Zhao et al., 2022). The diversity of components and the range of molecular structures significantly influence the bioactive encapsulation and delivery capabilities of oleogels (Chuesiang et al., 2022; Dhal et al., 2023; Dong et al., 2020).
II. For instance, the delivery of lipid-soluble components from the oleogel is influenced by the quantity of oleogelators, which determines the texture and structure of the oleogels (O'Sullivan et al., 2016; O'Sullivan et al., 2017). Chuesiang et al. (2022) formulated oleogels using hydroxypropyl methylcellulose (HPMC) of different grades. The HPMC grade with 4000 cp provided the highest viscosity oleogel, which better entrapped bioactive compounds and prevented droplet coalescence. However, this oleogel exhibited a lower release rate of free fatty acids and curcumin. Thus, even when using the same oleogelator, variations in its nature can influence the release ability of oleogels.
III. Oleogels show promise as carriers for nutraceuticals in food, and future studies should explore their ability to deliver lipid-soluble compounds like β-carotene, improving stability and appeal (Zhang, Cui, et al., 2022; Zheng et al., 2020).
IV. In vivo studies are needed to understand the health effects of oleogel-integrated margarine, particularly on lipid digestion, metabolism, and absorption, to ensure the safety and effectiveness of these ingredients in food (Dent et al., 2022; Günal-Köroğlu et al., 2024).
V. Creating oleogel-based emulsions could address the limitations of oleogels, allowing for the effective delivery of both lipophilic and water-soluble nutrients and broadening their applications in bioactive compound delivery (Santos et al., 2024).
VI. Heat application in oleogel formation can cause lipid oxidation, but using antioxidants helps reduce this. More research is needed on the specific oxidation products, especially from phenolics, and methods like temperature optimization and inert conditions could help address these challenges (Ramírez-Carrasco et al., 2024).
VII. Oleogelation can prevent oil leakage in microencapsulation, enhance oxidative stability, and protect sensitive bioactive compounds, expanding the potential uses of microencapsulated products (Günal-Köroğlu et al., 2024; O'Sullivan et al., 2017).
VIII. To justify the higher cost of oleogels in meat products, future research should focus on demonstrating their health benefits, improving sensory qualities, and enhancing shelf life, thereby offering added value to consumers (López-Pedrouso et al., 2021; Manzoor, Masoodi, Rashid, et al., 2022).
IX. Integrating essential oils into oleogels can address challenges like aroma and volatility, enhance stability, and improve shelf life, while also amplifying the antimicrobial and antioxidant properties of the products (Zhang, Chuesiang, et al., 2022).
X. Co-delivering hydrophobic and hydrophilic nutraceuticals in oleogels can improve stability, bioavailability, and efficacy, offering a versatile platform for complex formulations with targeted health benefits (Lu et al., 2022; Pawar et al., 2024).
XI. Recent study on lidocaine-containing emulsions suggest that SPAN80-stabilized oleogels offer better stability, moisture absorption, and favorable mechanical properties compared to TWEEN80-stabilized ones. Lidocaine does not significantly affect the stability of the emulsions, making it suitable for formulations without compromising physicochemical properties. These oleogels show promise for medical applications, particularly in pain relief and dermatological treatments, due to their stability, moisture retention, and improved skin application (Kudłacik-Kramarczyk et al., 2024).
XII. Another report examined the role of hydrocolloids and oleogels in improving the texture and quality of dairy products. Hydrocolloids like pectin, alginate, and carrageenan enhance dairy texture, and oleogels showed potential for future applications dairy foods to improve the sensorial and physicochemical properties. In addition, oleogel has broad prospects in dairy foods as a fat substitute (Zhang et al., 2024).
XIII. Research into using oleogels as a milk fat substitute in dairy products could lead to healthier alternatives, retaining desirable textures and sensory qualities while improving nutritional profiles. Finally, further investigations into the application of bioactive oil gel in the plant-based meat analogue and plant-based milk substitute industries are highly recommended.

## 6 The outlook and limitations

Oleogels have emerged as a promising fat substitute due to their capacity to transform liquid oils rich in unsaturated fatty acids into semi-solid structures, preserving nutritional quality while imparting the desirable functionality of traditional solid fats. This transformation is typically achieved by incorporating structuring agents—such as natural waxes, monoglycerides, fatty alcohols, or cellulose derivatives—into high-unsaturated oils like sunflower, olive, linseed, or fish oil (Martins et al., 2020; Patel & Dewettinck, 2016). These systems have been successfully applied across a variety of food matrices, including baked goods, margarine, chocolate, dairy spreads, and processed meats, enabling significant reductions in saturated and trans fatty acids (Nacak et al., 2021).

While oleogelation has emerged as a promising method for replacing trans and saturated fats in food products—driven by legislative restrictions, health concerns, and environmental issues related to palm fat—there are still notable limitations. Despite successful applications across various food categories and promising results in maintaining or improving product quality, challenges remain. These include the need for further development of oleogelation techniques and optimization of manufacturing processes. For oleogels to fully replace unhealthy fats on an industrial scale, more research is required to enhance their functionality, consistency, and scalability in diverse food systems (Pușcaș, Mureșan, Socaciu, & Muste, 2020).

The increasing health concerns associated with saturated and trans fats have placed oleogels at the forefront of fat reformulation strategies in the food industry (Tavernier et al., 2018). Their performance depends on factors such as gelator type and concentration, oil composition, and processing parameters like cooling rate and shear (Manzoor, Masoodi, Rashid, Naqash and Ahmad, 2022). These variables influence key functional properties, including oil-binding capacity, thermal stability, and texture—critical attributes when replicating the role of plastic fats in conventional applications.

In bakery products, oleogels help maintain desirable crumb structure and moisture while enhancing the lipid profile (Pușcaș et al., 2020). In meat systems, they can partially or fully replace animal fats without adversely affecting emulsion stability or cooking loss, although some sensory changes have been noted depending on the formulation (Ferro et al., 2021). Dairy applications, such as butter and cheese analogs, also benefit from the spreadability and oxidative stability offered by oleogel systems (Chowdhury et al., 2024).

Despite these advantages, challenges remain related to oxidative stability, sensory acceptance, cost-effectiveness, and scale-up feasibility. Nonetheless, with growing consumer demand for clean-label and healthier food options, oleogels offer a compelling avenue for reducing the health risks associated with conventional fats. Continued research should focus on optimizing formulation strategies and improving consumer perception to facilitate broader industrial adoption. In addition, the need for more systematic studies in this area to guide future research and industrial application.

## 7 Conclusions

Oleogels have gained considerable attention as a novel fat substitute in various food applications—including bakery products, margarine, meat products, and confectionery—due to their ability to reduce saturated and trans fat content while preserving the desirable solid-like texture of conventional fats. Importantly, oleogels maintain the chemical composition and nutritional value of the liquid oils from which they are structured, offering a healthier alternative without compromising product quality.

Beyond fat replacement, oleogels have emerged as versatile carriers for functional and bioactive compounds, such as polyphenols, omega fatty acids, vitamins, essential amino acids, proteins, and essential oils. This review critically evaluates recent advancements in oleogel formulation, characterization, and application, with a particular emphasis on their potential as delivery systems in food matrices. Studies have shown that oleogels not only enhance the stability and bioavailability of encapsulated compounds but also protect them from oxidative and digestive degradation—an essential attribute for preserving functionality in complex food systems.

Numerous reformulation efforts have demonstrated that incorporating oleogels into foods such as baked goods and meat products can achieve equal or superior techno-functional and physicochemical properties compared to conventional formulations, while concurrently enhancing nutritional profiles. Nevertheless, further optimization is needed in processing methods, selection of oleogelators, and gelation techniques to maximize functionality and industrial viability.

In conclusion, oleogelation offers a scientifically promising platform for both fat substitution and the controlled delivery of bioactives in foods. Its alignment with current trends in plant-based, clean-label, and health-oriented food innovation underscores its relevance for future applications. Continued research is essential to overcome current limitations, scale production processes, and fully integrate oleogels into the next generation of functional and sustainable food products.

## CRediT authorship contribution statement

**Taha Mehany:** Conceptualization, Methodology, Writing – original draft, Writing – review & editing, Data Curation, Formal analysis, Validation, Software, Investigation, Project administration, Funding acquisition, Supervision. **Oscar Zannou:** Writing – original draft, Formal analysis, Investigation. **Kouame F. Oussou:** Writing – original draft preparation. **Ifagbémi B. Chabi:** Writing – original draft preparation. **Reza Tahergorabi:** Data curation, Funding acquisition, Project administration, Visualization, Validation.

## Declaration of competing interest

The authors declare that they have no known competing financial interests or personal relationships that could have appeared to influence the work reported in this paper.

## Data Availability

Data will be made available on request.
